# Patterns of germline and somatic mutations in 16 genes associated with mismatch repair function or containing tandem repeat sequences

**DOI:** 10.1002/cam4.2702

**Published:** 2019-11-25

**Authors:** Shih‐Ching Chang, Yuan‐Tzu Lan, Pei‐Ching Lin, Shung‐Haur Yang, Chien‐Hsing Lin, Wen‐Yi Liang, Wei‐Shone Chen, Jeng‐Kai Jiang, Jen‐Kou Lin

**Affiliations:** ^1^ Division of Colon & Rectal Surgery Department of Surgery Taipei Veterans General Hospital Taipei Taiwan; ^2^ Department of Surgery Faculty of Medicine School of Medicine National Yang‐Ming University Taipei Taiwan; ^3^ Department of Clinical Pathology, Yang‐Ming Branch Taipei City Hospital Taipei Taiwan; ^4^ Department of Health and Welfare University of Taipei Taipei Taiwan; ^5^ National Yang‐Ming University Hospital Yilan Taiwan; ^6^ Division of Genomic Medicine National Health Research Institutes Zhunan Taiwan; ^7^ Department of Pathology Taipei Veterans General Hospital Taipei Taiwan

**Keywords:** colorectal cancer, EMAST, MMR, MSI, mutation

## Abstract

**Background:**

We assumed that targeted next‐generation sequencing (NGS) of mismatch repair‐associated genes could improve the detection of driving mutations in colorectal cancers (CRC) with microsatellite instability (MSI) and microsatellite alterations at selected tetranucleotide repeats (EMAST) and clarify the somatic mutation patterns of CRC subtypes.

**Material and methods:**

DNAs from tumors and white blood cells were obtained from 81 patients with EMAST(+)/MSI‐high (MSI‐H), 78 patients with EMAST(+)/microsatellite stable (MSS), and 72 patients with EMAST(−)/MSI‐H. The germline and somatic mutations were analyzed with a 16‐genes NGS panel.

**Results:**

In total, 284 germline mutations were identified in 161 patients. The most common mutations were in *EPCAM* (24.8%), *MSH6* (24.2%), *MLH1* (21.7%), and *AXIN2* (21.7%). Germline mutations of *AXIN2*, *POLE*, *POLD1*, and *TGFBR2* also resulted in EMAST and MSI. EMAST(+)/MSI‐H tumors had a significant higher mutation number (205.9 ± 95.2 mut/MB) than tumors that were only EMAST(+) or MSI‐H (118.6 ± 64.2 and 106.2 ± 54.5 mut/MB, respectively; both *P* < .001). In patients with *AXIN2* germline mutations, the number of pathological somatic mutations in the tumors was significantly higher than those without *AXIN2* germline mutations (176.7 ± 94.2 mut/MB vs 139.6 ± 85.0 mut/MB, *P* = .002).

**Conclusion:**

Next‐generation sequencing could enhance the detection of familial CRC. The somatic mutation burden might result from not only the affected genes in germline mutations but also through the dysfunction of downstream effectors. The *AXIN2* gene might associate with hypermutation in tumors. Further in vitro experiments to confirm the causal relationship is deserved.

## Graphical abstract

For patients with microsatellite instability or microsatellite alterations at selected tetranucleotide repeats, the somatic mutation burden occurs due to the dysfunction of downstream effectors but not the affected gene with germline mutation. The dysfunction of AXIN2 might affect both the canonical APC pathway and the hypermutation mechanism.

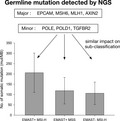

## 1 INTRODUCTION

Since 2006, colorectal cancer (CRC) has been the most common cancer in Taiwan, with an incidence of 44.8/100 000.^1^ In 2015, there were more than 15 000 new CRC patients diagnosed, with an annual incidence of 66.3/100 000.^2^ With the increasing incidence of CRC, several reports, including ours, found that approximately 15%‐20% of CRC patients have at least one first‐degree relative with the disease, and this type of disease is often considered familial.^3^, ^4^, ^5^

The most common of hereditary CRC syndrome, Lynch syndrome, is associated with deficient mismatch repair (MMR) genes.^6^, ^7^, ^8^ However, no germline mutations in MMR genes have been found in nearly half the patients with Lynch syndrome, as defined based on the Amsterdam criteria.^9^, ^10^, ^11^, ^12^ Additionally, there are several familial clusters of CRC in which no germline mutation in other genes could be identified.^13^, ^14^, ^15^ Traditionally, the identification of Lynch syndrome has been based on the Bethesda criteria or Amsterdam criteria, followed by microsatellite analysis by genotyping or immunohistochemistry of MMR proteins; based on which MMR protein is not expressed, germline mutations are investigated by Sanger sequencing.^16^ In addition to germline mutations of MMR genes, somatic mutations of MMR genes and hypermethylation promoter of *MLH1* have also been implicated as mechanisms underlying microsatellite instability (MSI).^17^ Even with extensive molecular analysis, some reports showed that no definite cause including germline mutations, somatic mutations or *MLH1* promoter hypermethylation could be found in nearly one‐third of patients with an abnormal MMR status or MSI and a Lynch syndrome‐consistent family history.^18^, ^19^

Another variant of MSI, elevated microsatellite alterations at selected tetranucleotide repeats (EMAST), possibly originating from *MSH3* dysfunction or inflammation, have been reported to have a unique phenotype in CRC.^20^, ^21^, ^22^, ^23^ *MSH3* dysfunction is a complex defect in cancer cells that not only generates EMAST but also may contribute to chromosomal instability and aneuploidy.^20^ EMAST(+) cancers share features with MSI+ CRCs^24^; however, evidence has shown conflicting survival results of stage II/III CRC patients after adjuvant 5‐FU‐based chemotherapy regardless of EMAST status^25^ compared to the survival results of patients with MSI‐high (MSI‐H) tumors.^26^ Investigations of the relationships between EMAST and chemotherapy response using targeted gene mutation analysis to gain insight into the mechanisms related to inflammation‐induced compartmentalization and inactivation of *MSH3* are ongoing.

The technique of next‐generation sequencing (NGS) has improved quickly, and a number of new disease genes, including both rare and common variants, have been identified through this technique.^27^, ^28^, ^29^ The clinical use of NGS is becoming feasible because the associated expense has dramatically decreased. Several extensive analyses of Lynch‐like syndrome by NGS showed the association of MMR deficiency with mutations or epimutations of several other genes, including *POLD1*, *POLE*,^30^, ^31^ and *AXIN1/2*.^32^ In addition, a high tumor mutation burden has been reported in CRC with dMMR or MSI.^33^ To further investigate the germline and somatic mutation of MMR‐associated genes, we enrolled patients with EMAST and patients with MSI, and analyzed the tumor mutation burden and germline mutations with a 16 MMR‐associated gene panel using NGS.

## 2 MATERIALS AND METHODS

### 2.1 Ethics statement

The study was approved by the Institutional Review Board of Taipei Veterans General Hospital (number 2017‐06‐004BC) and the data were analyzed anonymously.

### 2.2 Patient sample collection

We obtained information on 81 patients with EMAST(+) and MSI‐H CRC, 78 patients with EMAST(+) and microsatellite stable (MSS) CRC, and 72 patients with EMAST(−) but MSI‐H CRC from a database of 1505 CRC patients. Another 60 patients without EMAST(+) or MSI‐H CRC were randomly selected from the database. In total, DNA samples from 291 paired tumor and white blood cells samples (WBCs) were obtained from the Biobank of Taipei Veterans General Hospital.

This prospectively collected clinical database consisted of 1505 patients with CRCs who received surgery at the Taipei Veterans General Hospital between 2000 and 2010.^34^, ^35^, ^36^ This database prospectively collected clinical information including age, sex, personal and family medical histories, tumor locations, and CEA levels. The pathological data consisted of TNM stage, differentiation, mucinous histology, and the presence of inflammation in the stroma and lymphovascular invasion. The exclusion criteria were as follows: death within 30 days of surgery, preoperative radiochemotherapy, or emergency operations. The molecular data, including microsatellite status, EMAST status, and 12 genes, including 139 hot spots, were analyzed by MassArray.^34^, ^36^ The protocols for analyzing MSI, EMAST, and somatic mutations were the same as those used in previous studies.^24^, ^34^, ^36^ The MSI markers consisted of D5S345, D2S123, BAT25, BAT26, and D17S250. The five tetranucleotide markers included D20S82, D20S85, D8S321, D9S242, and MYCL1 to determine the EMAST status. Both MSI‐H and EMAST(+) were defined as at least two positive markers out of the aforementioned five markers. The twelve genes analyzed by MassArray were *APC*, *FBXW7*, *TP53*, *KRAS*, *NRAS*, *HRAS*, *BRAF*, *PI3KCA*, *PTEN*, *AKT1*, *TGFBR2*, and *SMAD4*.

### 2.3 Next‐generation sequencing

We used the high‐throughput genome sequencer Illumina HiSeq2500 system, to comprehensively explore the DNA sequences of all exons of 16 well‐known DNA repair‐related genes, namely, *AXIN1*, *AXIN2*, *BAX*, *CTNNB1*, *EPCAM*, *EXO1*, *MLH1*, *MSH2*, *MSH3*, *MSH6*, *PCNA*, *PMS1*, *PMS2*, *POLD1*, *POLE*, and *TGFBR2*, which were selected based on previous studies.^37^, ^38^ A total of 100 ng of DNA from each individual was used to construct a sample library using a Roche KAPA Library Preparation Kit (Roche). Each DNA sample was fragmented and used to prepare the DNA library by performing end‐repairing, a‐overhang addition, adaptor ligation, and size selection (150‐350 bp). The target DNA of the DNA repair‐related genes was enriched using the probe‐based methods. The probes were synthesized by Integrated DNA Technologies (USA) according to our previously designed probe sequences, and the capture procedure was performed following the IDT guidelines. After the probe‐based enrichment, a library of each pool was amplified with 14 cycles. The amplified libraries were quantified using a qPCR system and pooled into a new 1.5‐mL tube as a 10 nmol/L pooled DNA library. The final pool was used for sequencing (illumina HiSeq2500 sequencer, 2 × 100 bp). The raw output of each individual sample was 250 Mb, and the average depth of the target regions was >1000×. The sequence of each read was trimmed based on the quality score (Q30), and any read less than 45 bp in length was discarded before the following analysis. Reads were aligned to the human hg19 reference genome using BWA‐MEM (http://bio-bwa.sourceforge.net/), and the GATK Unified Genotyper (GATKLite version 2.3‐9) was used for calling variants. After variant calling, we used Variant Effect Predictor (http://grch37.ensembl.org/Homo_sapiens/Tools/VEP) to annotate the identified variants for the subsequent statistical analysis. The mean read depth was 2404.2 ± 1457.7 and 4119.4 ± 2991.1 in the germline and somatic mutation analyses, respectively.

### 2.4 Statistical analysis

A chi‐square test and two‐tailed Fisher's exact test were used to compare the genotype frequencies of the clinicopathological features. Numerical values were compared using Student's *t* test and one‐way ANOVA. The data are expressed as the mean ± standard deviation. Statistical significance was defined as *P* < .05. Statistical analyses were performed using SPSS for Windows (version 16.0).

## 3 RESULTS

### 3.1 Clinicopathological features of patients with/without germline mutations

As shown in Table 1, patients with germline mutations had a significantly higher frequency of a proximal tumor location and stage I and II disease. The other pathological features were similar between patients with/without germline mutations.

**Table 1:** Comparison of clinicopathological features of 231 patients carrying tumors either EMAST(+) or MSI‐H with/without germline mutation

Clinicopathological features	Without GM (n = 70) (%)	With GM (n = 161) (%)	*P* value
Age	70.8 ± 14.1	67.9 ± 13.1	.456
Male Gender	37 (52.9)	96 (59.6)	.441
Location			
Proximal colon	21 (30.0)	78 (48.4)	.003^a^
Distal colon	30 (42.9)	35 (21.7)	
Rectum	19 (27.1)	48 (29.8)	
Poor differentiation	9 (12.9)	23 (14.3)	.773
Lymphovascular invasion	11 (15.7)	36 (22.4)	.329
Mucinous Histology	11 (15.7)	24 (14.9)	.875
TNM stage			
I	17 (15.7)	16 (9.9)	.011^a^
II	23 (32.9)	81 (50.3)	
III	19 (27.1)	46 (28.6)	
IV	11 (15.7)	18 (11.2)	

Abbreviation: GM, germline mutation.

a chi‐square test with statistically significant difference, *P* < .05.

### 3.2 Patterns and frequency of germline mutations

In 231 patients with either EMAST(+) or MSI‐H CRC, 284 pathological germline mutations (3 deletion, 18 insertion frameshift and 263 missense variants) were identified in 161 patients. Seventy patients had no germline mutation. Of the 60 patients without EMAST(+) or MSI‐H CRC, we did not find any germline mutations using this 16‐gene panel. Therefore, the panel for 16 DNA repair‐related genes panel was estimated to detect pathologic germline mutations in at least 10.6% of the patients (161 of 1505 patients) in this database. The other 70 patients with either EMAST(+) or MSI‐H tumors with clinical suspected Lynch syndrome, but no germline mutations identified through genetic testing were proposed as having Lynch‐like syndrome.^39^ In addition to germline mutations of MMR genes, Lynch‐like syndrome could result from somatic mutations of MMR genes or methylation of the *MLH1* promotor.^17^ We analyzed the somatic mutations of five MMR genes (*MLH1*, *MSH2*, *EPCAM*, *MSH6*, *PMS2*) in the 70 patients with Lynch‐like syndrome and found 68 somatic mutations in 34 patients (Supplemental Excel). Our study did not obtain information on *MLH1* promoter methylation, but we found 18 *BRAF* ^V600E^ mutations (9 had somatic mutations of MMR genes) in 70 patients with Lynch‐like syndrome. Because *BRAF* mutations are correlated with *MLH1* methylation,^40^, ^41^ we could assume that sporadic MSI tumors could develop through *MLH1* promoter methylation and somatic MMR mutations to induce MSI carcinogenesis.

**Figure 1:**
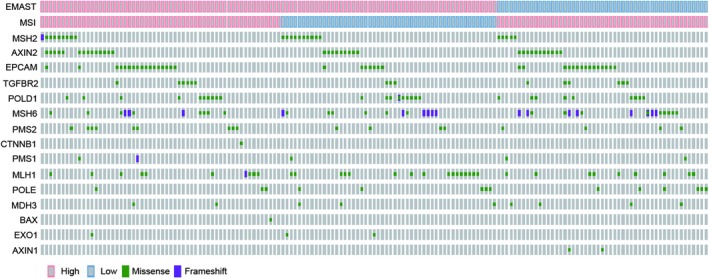
Distributions of 16 MMR‐related gene mutations in 161 cases with germline mutation

In 161 patients with CRC with germline mutations, the most commonly mutated genes were *EPCAM* (40, 24.8%), *MSH6* (39, 24.2%), followed by *MLH1* (35, 21.7%), *AXIN2* (35, 21.7%), *POLD1* (28, 17.4%), and *MSH2* (24, 14.9%). Fifteen patients had two mutations in the same gene (5 *AXIN2*, 1 *MLH1*, 6 *MSH2*, 1*MSH6*, and 2 *POLD1*). No mutation was noted in *PCNA* (Table 2; Figure 1).

**Table 2:** Distributions of 16 gene mutations in 161 patients with germline mutation and their associations with EMAST and MSI subtypes

Gene	Mutation no.	Case No. (%) n = 161	EMAST(+) (%) n = 110	MSI‐H (%) n = 109
*AXIN1*	2	2 (1.2)	—	—
*AXIN2*	40	35 (21.7)	24 (68.6)	26 (74.3)
*BAX*	1	1 (0.6)	—	—
*CTNNB1*	1	1 (0.6)	—	—
*EPCAM*	40	40 (24.8)	24 (60.0)	33 (82.5)
*EXO1*	3	3 (1.3)	—	—
*MLH1*	36	35 (21.7)	26 (74.3)	19 (54.3)
*MSH2*	30	24 (14.9)	19 (79.2)	14 (58.3)
*MSH3*	9	9 (5.6)	5 (55.6)	5 (66.7)
*MSH6*	40	39 (24.2)	23 (59.0)	27 (69.1)
*PCNA*	0	0 (0.0)	—	—
*PMS1*	5	5 (3.1)	—	—
*PMS2*	17	17 (10.6)	13 (76.5)	13 (76.5)
*POLD1*	30	28 (17.4)	19 (67.9)	19 (67.9)
*POLE*	15	15 (9.3)	7 (46.7)	10 (66.7)
*TGFBR2*	15	15 (9.3)	10 (66.7)	12 (80.0)

### 3.3 Germline mutations associated with EMAST and MSI genotypes

Studies demonstrated that in patients with Lynch syndrome with dysfunctional MMR genes, 90% had MSI.^8^, ^17^ As shown in Table 2, we found that the majority of individual germline gene mutations have different impacts on the EMAST or MSI genotypes. From our results, we could conclude that the majority of EMAST and MSI result from not only MMR dysfunction but also germline mutations of *AXIN2*, *POLE*, *POLD1*, and *TGFBR2*. These findings need to be confirmed in an in vitro study.

In 161 CRC patients with germline mutations, the mutation number ranged from 1 to 4; 81 patients had a single germline mutation, 47 patients had two germline mutations, 23 patients had three germline mutations, and 10 patients had four germline mutations (Table 3). According to the EMAST and MSI tumor classifications, patients with germline mutations had similar frequencies of these three molecular subtypes. EMAST(+) with MSI‐H:36.0%, EMAST(+) with MSS: 32.3%, and EMAST(−) with MSI‐H: 31.7%, *P* = .856). Additionally, the number of germline mutations was not associated with the EMAST or MSI classification (Table 3).

**Table 3:** The number of germline mutation was not associated with EMAST and MSI subtypes

No. of GM	Case no.	EMAST(+) MSI‐H (%)	EMAST(+) MSS (%)	EMAST(−) MSI‐H (%)	*P* value
0	70	23 (32.9)	24 (34.2)	23 (32.9)	.876
1	81	30 (37.0)	28 (34.6)	23 (28.4)	
2	47	15 (31.9)	15 (31.9)	17 (36.2)	
3	23	7 (30.4)	8 (34.8)	8 (34.8)	
4	10	6 (60.0)	1 (10.0)	3 (30.0)	
≥1	161	58 (36.0)	52 (32.3)	51 (31.7)	.856

Abbreviation: GM, Germline mutation.

### 3.4 Patterns and frequencies of somatic mutations

There were 3083 somatic mutations, including 434 5′ UTR variants, 540 splice region variants and 290 synonymous variants in the coding region in patients with either EMAST(+) or MSI‐H tumor. Among these somatic mutations, 1819 pathological mutations (654 frameshift, 1004 missense, 1 start‐loss, and 160 nonsense variants) were found, including 1374 mutations in 161 patients with germline mutations and 445 mutations in 70 patients without germline mutations. After the elimination of the 284 germline mutations, there were 1090 somatic mutations in 161 patients with germline mutations. All total numbers of somatic mutations and numbers of pathological somatic mutations per tumor were similar between patients with and without germline mutations (270.7 ± 125.1 mut/MB vs 257.8 ± 111.4 mut/MB, *P* = .454; 146.1 ± 89.0 mut/MB vs 143.2 ± 83.6 mut/MB, *P* = .820, Table 3). Additionally, tumor with different numbers of germline mutations had similar numbers of somatic mutations (*P* = .902, Table 4).

**Table 4:** Numbers of all and pathological somatic mutation per colorectal tumor in 161 patients with different germline mutation numbers

No. of GM	Case no.	No. of All SM (per tumor)	*P* value	No. of PSM (per tumor)	*P* value
0	70	257.8 ± 111.4	0.902	143.2 ± 83.6	.982
1	81	269.4 ± 114.7		150.5 ± 83.5	
2	47	264.5 ± 146.4		138.6 ± 99.6	
3	23	281.3 ± 81.3		145.0 ± 71.0	
4	10	287.1 ± 186.8		148.0 ± 124.1	
≥1	161	270.7 ± 125.1	0.454	146.1 ± 89.0	.820

Abbreviations: GM, germline mutation; SM, somatic mutation; PSM, pathological somatic mutation.

The downstream numbers of somatic mutations were associated with the EMAST‐MSI subtypes. Among all patients with or without germline mutations, EMAST(+) and MSI‐H tumors had a mean of 205.9 ± 95.2 somatic mut/MB, which was significantly higher than the mean number of somatic mutations in tumors that were only EMAST(+) or only MSI‐H (118.6 ± 64.2, 106.2 ± 54.5 mut/MB, respectively; both *P* < .001, Table 5). The somatic mutation patterns in EMAST and MSI subtype tumors were similar between patients with and without germline mutations (Table 6). The genes with higher numbers of somatic mutations were *AXIN2*, *BAX*, *MSH3*, *MSH6*, *PMS1*, *POLD1 POLE*, and *TGFBR2*. In particular, the *MSH3* mutations were almost located in EMAST(+) and MSI‐H tumors. As shown in the Table 5, the somatic mutation frequencies of individual genes, except *PMS1*, were higher in EMAST(+) and MSI‐H tumors than in other subtypes of tumors.

**Table 5:** The numbers of downstream somatic mutation were associated with EMAST‐MSI subtypes

	Total	EMAST(+) MSI‐H	EMAST(+) MSS	EMAST(−) MSI‐H
With GM				
Case no.	161	58	52	51
No. of SM (mut/MB)	146.1 ± 89.1	212.0 ± 100.5	122.3 ± 64.0	106.9 ± 54.5
Without GM				
Case no.	70	23	24	23
No. of SM (mut/MB)	143.2 ± 83.6	195.9 ± 107.6	121.9 ± 53.4	112.4 ± 54.5
All patients				
Case no.	231	81	76	74
No. of SM (mut/MB)	—	205.9 ± 95.2^a^, ^b^	118.6 ± 64.2^a^	106.2 ± 54.5^b^

Abbreviations: GM, germline mutation; SM, somatic mutation.

a Statistically significant difference (*P* < .001).

b Statistically significant difference (*P* < .001).

**Table 6:** The somatic mutation patterns of EMAST and MSI subtypes between cases with and without germline mutations

No. of SM (%)	With GM (n = 161)			Without GM (n = 70)		
Gene	EMAST(+) MSI‐H (n = 58)	EMAST(+) MSS (n = 52)	EMAST(−) MSI‐H (n = 51)	EMAST(+) MSI‐H (n = 23)	EMAST(+) MSS (n = 24)	EMAST(−) MSI‐H (n = 23)
*AXIN1*	21 (36.2)	2 (3.8)	1 (2.0)	2 (8.7)	1 (4.2)	0 (0.0)
*AXIN2*	26 (44.8)	6 (11.5)	5 (9.8)	9 (39.1)	6 (25.0)	5 (21.7)
*BAX*	48 (82.8)	19 (36.5)	16 (31.4)	18 (78.3)	12 (50.0)	9 (39.1)
*CTNNB1*	2 (3.4)	4 (7.7)	3 (5.9)	3 (13.0)	0 (0.0)	0 (0.0)
*EPCAM*	7 (12.1)	6 (11.5)	2 (3.9)	9 (39.1)	2 (8.3)	1 (4.3)
*EXO1*	17 (29.3)	2 (3.8)	2 (3.9)	4 (17.4)	2 (8.3)	3 (13.0)
*MLH1*	16 (27.6)	5 (9.6)	5 (9.8)	4 (17.4)	4 (16.7)	2 (8.7)
*MSH2*	15 (25.9)	8 (15.4)	3 (5.9)	2 (8.7)	1 (4.2)	6 (26.1)
*MSH3*	55 (94.8)	2 (3.8)	4 (7.8)	19 (82.6)	2 (8.3)	3 (13.0)
*MSH6*	35 (60.3)	7 (13.5)	14 (27.5)	19 (82.6)	6 (25.0)	4 (17.4)
*PCNA*	1 (1.7)	1 (1.9)	0 (0.0)	0 (0.0)	0 (0.0)	0 (0.0)
*PMS1*	91 (156.9)	102 (196.2)	100 (196.1)	51 (221.7)	42 (175.0)	43 (187.0)
*PMS2*	16 (27.6)	5 (9.6)	3 (5.9)	4 (17.4)	2 (8.3)	2 (8.7)
*POLD1*	52 (89.7)	19 (36.5)	10 (19.6)	10 (43.5)	5 (20.8)	6 (26.1)
*POLE*	64 (110.3)	44 (84.6)	21 (41.2)	14 (60.9)	22 (91.7)	10 (43.5)
*TFGBR2*	91 (156.9)	54 (103.8)	58 (113.7)	36 (156.5)	25 (104.2)	23 (100.0)

Abbreviations: GM, germline mutation; SM, somatic mutation.

If we do not consider the rare mutations in *CTNNB1* (1 patient) and *EXO1* (3 patients), tumors carrying germline mutations of *AXIN2* had the highest somatic mutation number in this 16‐gene panel. The mutation frequencies of the other genes are shown in Table S1. In patients carrying *AXIN2* germline mutations, the total number of somatic mutations and the number of pathological somatic mutations in tumors were 315.5 ± 122.4 and 176.7 ± 94.2 mut/MB, respectively. Both numbers were significantly higher than those in patients without *AXIN2* germline mutations (258.2 ± 119.0 mut/MB, *P* = .010; 139.6 ± 85.0 mut/MB, *P* = .020), indicating that dysfunction of *AXIN2* could induce the hypermutating status in tumors.

### 3.5 Identification of functional solitary germline mutations

Of the 81 single‐gene mutations in patients with germline mutations, 40 patients had rare single‐gene mutation, including two *AXIN1* mutations, 12 *AXIN2* mutations, one *BAX* mutation, one *CTNNB1* mutation, two *MSH3* mutations, two *PMS1* mutations, four *POLD1* mutations, nine *POLE* mutations, and seven *TGFBR2* mutations. In addition to five major MMR genes (*MLH1*, *MSH2*, *EPCAM*, *MSH6*, and *PMS2*) and three additional genes with rare mutations (*AXIN1*, *BAX*, and *CTNNB1*), another seven genes were included to identify the associated gene mutations, which are shown in Table S2 and Figure S1. Eleven (31.4%) *AXIN2* mutations, eight (53.3%) *POLE* mutations and six (40%) *TGFBR2* mutations could result in the MSI‐H or EMAST(+) phenotypes without any accompany germline mutations of 16 MMR‐related genes. The molecular and clinicopathological features of solitary *AXIN2*, solitary *TGFBR2* and solitary *POLE* were demonstrated in Figure S2. The majority of *EXO1*, *MSH3*, *PMS1*, and *POLD1* mutations might need other accompanying MMR‐related germline gene mutations to result in the necessary molecular changes, including MSI‐H, EMAST(+) or somatic mutations. Therefore, mutations in *AXIN2*, *POLE*, and *TGFBR2* might be driving mutations of familial colon cancer syndrome or there may be other genes involved we did not detect.

### 3.6 Rare germline mutations

Four patients had rare germline mutations in *AXIN1* (2 patients), *BAX* (1 patient), and *CTNNB1* (1 patient). The clinicopathological features are shown in Table S3. These tumors did not have mucinous histology or lymophovascular invasion.

## 4 DISCUSSION

This study provided several major contributions to the knowledge of familial CRC. First, targeted sequencing by NGS could increase the detection of familial CRC including Lynch syndrome. Second, the somatic mutation burden occurs due to the dysfunction of downstream effectors but not the affected gene with germline mutation. Third, the dysfunction of *AXIN2* might affect both the canonical APC pathway and the hypermutation mechanism. Fourth, we demonstrated the similarities and differences between the clinicopathological features of patients with CRC with and without germline mutations. Fifth, we described the characteristics of patients with rare germline mutations.

Our series showed that the incidence of familial CRC with identified germline mutations was as high as 10%. Of these 161 patients, 100 patients (62.1%) fulfilled the Bethesda criteria, which was a lower percent than was found in another Asian study.^42^ The major reason for this lower percent was inadequate or inaccurate of personal and family histories. This limitation has been reported previously.^43^, ^44^ Considering the five commonly mutated MMR genes associated with Lynch syndrome, namely, *MLH1*, *MSH2*, *EPCAM*, *MSH6*, and *PMS2*, the incidence of detected Lynch syndrome was higher up to 7.97% (120 in 1505 patients had at least one mutation in these five MMR genes). This result was higher than in previous studies (3%‐6%), including ours.^4^, ^17^, ^42^, ^45^, ^46^, ^47^, ^48^, ^49^ The traditional method of Lynch syndrome screening was the application of the revised Bethesda criteria, detection of MSI or IHC of MMR proteins and then sequencing. Recently, with the advent of NGS, germline testing has progressively moved from mutational analysis of single genes toward a multigene panel analysis that could enhance the detection rate of Lynch syndrome. The other causes of the higher incidence of Lynch syndrome in our series were that we included patients with EMAST phenotypes and target sequences of MMR‐associated genes, including a high frequency of *EPCAM* mutations.

Seventy patients were not found to have germline mutations but had MSI or EMAST. We found that 23.1% had *BRAF* mutations and 50.0% (34/68) had somatic MMR mutations. Patients with CRC with somatic MMR mutations also had MSI.^18^ As *BRAF* mutations are correlated with *MLH1* methylation,^40^, ^41^ we could assume that sporadic MSI tumors could occur through *MLH1* methylation and somatic MMR mutations, resulting in MSI carcinogenesis.

Eight patients had more than two germline mutations and were considered to have constitutional mismatch repair deficiency syndrome (biallelic germline mutations). Apart from an earlier age of onset of CRC (32.1 ± 13.1), the other characteristics of these patients were identical to those of patients with Lynch syndrome. A previous study showed that patients with biallelic germline mutations had often had several tumors, starting in childhood.^50^ We could not confirm this in our patients because we did not have the relevant information in our database.

EMAST result in tetranucleotide instability, and the detection of EMAST and MSI‐H was defined as the presence of instability in at least two of the five markers. A previous study demonstrated the association between EMAST and the loss of *MSH3* nuclear expression in CRC.^20^, ^51^ Further, an in vitro study provided the evidence that *MSH3* knockdown could increase dinucleotide or tetranucleotide instability.^23^ However, our germline analysis demonstrated that only 4.5% (5/110) of EMAST patients had *MSH3* germline mutations, all of which were missense variants. We did not have data on *MSH3* nuclear expression. We could not conclude that these missense variants are associated with the loss of *MSH3* nuclear expression and the induced EMAST phenomenon. Because of the low frequency of *MSH3* germline mutations, we believe that the role of *MSH3* is limited in the EMAST phenomenon, which conflicts with the results of other reports.^20^, ^23^, ^51^

Furthermore, we found that patients with EMAST and MSI had higher somatic mutation rates with/without germline variants. Our result is reasonable because if mononucleotide, dinucleotide, and tetranucleotide frameshift mutations are not corrected, the underlying dysfunction of MMR proteins or other DNA repair‐associated proteins is more severe than in situations in which only tetranucleotide frameshifts or mono‐ or dinucleotide frameshifts are not corrected. In the patients without germline mutations, the number of *POLE* somatic mutations (60.9%) in EMAST(+)‐MSI‐H tumors was lower than in EMAST(+)MSS tumors (91.7%), but higher than in EMAST(−)‐MSI‐H tumors (43.5%). This result implies that EMAST, but not MSI, trigger *POLE* somatic mutations. For patients with germline mutations, *PMS1* somatic mutations were as high as 156.9% in EMAST(+)‐MSI‐H tumors, but lower than those in EMAST(+)‐MSS (196.2%) and EMAST(−)MSI‐H tumors(196.1%). This result means that the *PMS1* somatic mutation burden does not occur through the EMAST or MSI mechanism but rather through the dysfunction of genes with germline mutations.^52^, ^53^ Until now, there was no report linking EMAST and the tumor mutation burden. Our results deserve further study.

Our results, similar to those of other studies,^54^, ^55^ demonstrated that the tumors with EMAST or MSI were located predominantly in the proximal colon and had mucinous histology or poor differentiation but were diagnosed at an earlier stage, most commonly in stage II. These specific phenotypes were considered to be the result of MSI that cause a change in downstream genes rather than of the mechanism that induced the MSI. A previous study confirmed that hereditary MSI patients had better outcomes due to earlier stage disease at diagnosis compared to sporadic MSI patients.^56^

For the solitary *AXIN2*, *TGFBR2*, and *POLE* germline mutations, our previous study collected a database of CRC‐associated gene mutations. We deciphered the molecular and clinicopathological features of patients with solitary *AXIN2*, *TGFBR2*, and *POLE* germline mutations (Figure S2). In the solitary *AXIN2* germline mutation patients, their mutation loci were all located in the GSK3b, β‐catenin and PP2Ac‐binding domains. Furthermore, the tumors had several *APC*, *TP53*, and *KRAS* mutations. Additionally, a previous study suggested that wild‐type *AXIN2* inhibits the Wnt signaling pathway; however, mutated *AXIN2* dominantly activates Wnt signaling in cancer cells.^57^ These results implied that *AXIN2* might be an initiator of the canonical pathway through dysfunction of the β‐catenin destruction complex. More interestingly, germline mutations of *AXIN2* had a high tumor mutation burden in our results. As in a previous study, this partly explained the fact that *AXIN2* germline mutation are responsible for hereditary cancer without known molecular causes.^58^ Although *AXIN2* explained some causes of MSI and the EMAST phenomenon, the somatic *AXIN2* mutation rate was not very high. Only 24.7% of tumors had somatic *AXIN2* mutations. Because the *AXIN2* gene containing short coding oligonucleotide repeats, somatic mutations in the *AXIN2* gene have been reported in MSI CRC,^59^ but the functional consequences of these *AXIN2* changes remain unclear. Therefore, functional studies of the effects of *AXIN2* mutations on the canonical and MMR pathway deserve further study.

Based on a high mutation frequency in coding mononucleotide tracts,^60^ *TGFBR2* was considered to be the MSI CRC target gene.^61^ Indeed, our results showed that all 231 patients with EMAST or MSI had somatic mutations in the *TGFBR2* gene (Table 5). A previous study presented the evidence that familial CRC originated partly from germline *TGFBR2* mutations.^62^ However, another study did not find germline *TGFBR2* mutation associated with patients with early‐onset CRC or HNPCC.^63^ We still found 15 patients with *TGFBR2* germline mutations and six patients with solitary *TGFBR2* germline mutations. Three of these six patients with *TGFBR2* germline mutations had the same second‐hit mutation in *TGFBR2* (c.382_383delAA) in their tumors and these patients had similar clinicopathological features, including proximal tumor locations, early TNM stages and the EMAST(+)‐MSI‐H subtype (Figure S2). In the other three patients, the second‐hit mutation was in other loci of *TGFBR2*, and the clinicopathological features were different except for the presence of MSI‐H. These six germline mutation loci are all located in the intracellular and protein kinase genes. A recent study showed that cell lines harboring *TGFBR2* mutants failed to respond to exogenous TGF‐β stimulation, and re‐expression of wild‐type *TGFBR2* restored canonical TGF‐β signaling and proliferative inhibition, confirming the mutational loss of TGF‐β tumor suppressive activity.^64^ Therefore, germline *TGFBR2* mutations could be one of the causes of familiar CRC without MMR dysfunction.

Patients with/without *POLE* germline mutations had similar number of total and pathological somatic mutations in their tumors. Eight patients had solitary germline *POLE* mutations. Six of those eight patients had a second‐hit *POLE* mutation in their tumors. These eight patients did not have unique clinicopathological features, but had mutations in the EGFR signaling pathway, including mutations in *KRAS*, *BRAF*, and *PIK3CA* (6/8) (Figure S2). A recent study showed that hypermutant cancers were enriched for defects in mismatch repair pathway genes *POLE* and *POLD1*.^65^ Different *POLE* germline mutations could result in different mutation burdens in tumors and that not all tumors with *POLE* mutations were associated with hypermutation; drive mutations of *POLE* were uncovered outside the exonuclease domain, indicating that other domains may be responsible for proof reading.^65^ In our study, only two of eight solitary germline mutations were located in the exonuclease domain. This might explain the similar mutation burdens in tumors in patients with/without *POLE* germline mutations.

There were several advantages to this study. First, we extracted MSI or EMAST patients from a large database with detailed clinicopathological features and molecular signatures. Second, the study design involved somatic and germline mutation analysis. Third, we analyzed 16 MMR‐associated genes via NGS with high read depth, looking for both germline and somatic mutations. However, the lack of immunohistochemistry to support the loss or dysfunction of proteins induced by the mutations is the major limitation of this study.

Finally, our results provided evidence that NGS could enhance the detection rate of Lynch syndrome, which was underestimated in the past. The somatic mutation burden might occur through the dysfunction of downstream effectors rather than directly through the affected genes. We also identified several genes with germline mutations that may explain the familiar CRC. The *AXIN2 g*ene has dual functions in the canonical pathway and is associated with hypermutation in tumors. Our findings deserve to do in vitro experiments to confirm this causal relationship.

## CONFLICT OF INTERESTS

All the authors declare no competing interests, financial or non‐financial, in relation to the work described.

## AUTHOR CONTRIBUTIONS

Yuan‐Tzu Lan involved in study design and writing – original draft. Shih‐Ching Chang involved in study design and writing – review and editing. Pei‐Ching Lin and Chien‐Hsing Lin involved in molecular analysis including MSI, EMAST, and NGS. Wen‐Yi Liang analyzed the pathological slides. Jeng‐Kai Jiang, Wei‐Shone Chen, Shung‐Haur Yang, and Jen‐Kou Lin collected clinical data and samples.

## Data Availability

The datasets generated and/or analyzed during this study are available for noncommercial use from the corresponding author on reasonable request.
