# Supplementary material for: Patterns of germline and somatic mutations in 16 genes associated with mismatch repair function or containing tandem repeat sequences
Source: Cancer Med. 2019 Nov 25;9(2):476–86. doi: 10.1002/cam4.2702 (PMC6970039; doi:10.1002/cam4.2702)
Supplement: Supplementary file 1 [file CAM4-9-476-s001.pdf]

Figure S1. The associated genetic mutations of *AXIN2*, *EXO1*, *MSH3*, *PMS1*, *POLD1*, *POLE*, and *TGFBR2*

accompany gene	*AXIN2*
AXIN2	5
EPCAM	5
EXO1	1
MLH1	6
MSH2	5
MSH3	2
MSH6	7
PMS1	1
POLD1	3
POLE	1
TGFBR	1
no other mutation	11

accompany gene	*EXO1*
AXIN2	1
EPCAM	1
MLH1	1
MSH2	1
MSH6	1
PMS2	1

accompany gene	*MSH3*
AXIN2	2
EPCAM	2
MLH1	1
MSH2	2
MSH6	2
PMS2	2
POLD1	2
POLE	1
no other mutation	1

accompany gene	*PMS1*
AXIN2	1
EPCAM	2
MLH1	2
MSH2	3
PMS2	1
POLE	1
no other mutation	1

accompany gene	*POLD1*
AXIN2	3
EPCAM	4
MLH1	5
MSH2	2
MSH3	2
MSH6	10
POLD1	2
POLE	2
TGFBR	4
no other mutation	4

accompany gene	*POLE*
AXIN2	1
MLH1	2
MSH2	2
MSH3	1
MSH6	1
PMS1	1
PMS2	1
POLD1	2
no other mutation	9

accompany gene	*TGFBR2*
AXIN2	1
EPCAM	3
MLH1	1
MSH6	5
POLD1	4
no other mutation	6
