# Supplementary material for: Patterns of germline and somatic mutations in 16 genes associated with mismatch repair function or containing tandem repeat sequences
Source: Cancer Med. 2019 Nov 25;9(2):476–86. doi: 10.1002/cam4.2702 (PMC6970039; doi:10.1002/cam4.2702)
Supplement: Supplementary file 2 [file CAM4-9-476-s002.pdf]

Figure S2. Molecular and clinicopathological features of solitary germline mutation of *AXIN2*, *TGFBR2*, *POLE*

Molecular and clinicopathological features of Solitary *AXIN2* germline mutation

Sample no.	Germline Variants	Age	Gender	location	TNM	LVI	mucinous histology	Molecular alterations in tumor			
1142	R714W	77.82	male	Proximal	2	no	no	EMAST+MSI+	APC c.4348C>T		
1240	A417V	54.61	Male	rectum	3	yes	no	EMAST+MSS	APC c.850C>T	FBXW7 c.1745C>T	TP53 c.916C>T
1604	A603P	66.21	Male	proximal	2	no	no	EMAST+MSI+	APC c.C694C>T	TP53 c.586C>T	
2574	A603P	62.42	Female	Proximal	2	no	yes	EMAST+MSI+	KRAS c.38G>A	TGFBR2 c.382_383delAA	PIK3CA c.3140A>G
3182	G601A	77.46	Male	rectum	2	no	no	EMAST+MSS	APC c.3916G>T		
3262	D320V	47.89	Male	rectum	4	yes	no	EMAST-MSI+	KRAS c.35G>A	NRAS c.C181A	NRAS c182A>T
3324	A603P	67.95	Male	Distal	2	no	no	EMAST-MSI+			
3490	G601A	51.16	Female	rectum	2	no	no	EMAST-MSI+	KRAS c.35G>A	APC c.3340C>T	
3606	A417V	81.16	Male	proximal	3	yes	no	EMAST+MSI+	BRAF c.1799T>A		
3742	A417V	79.81	Female	rectum	2	no	no	EMAST-MSI+	KRAS c.35G>A	PTEN c.494G>A	
3956	A603P	71	Male	proximal	2	no	no	EMAST+MSI+	BRAF c.1799T>A	TGFBR2 c.373_374insA	

Molecular and clinicopathological features of Solitary *TGFBR2* germline mutation

Sample no.	Germline Variants	Age	Gender	location	TNM	differentiation	LVI			Molecular alterations in tumor
1110	V216I	69.49	male	proximal	2	mod	no	EMAST+MSI+	BRAF c.1799T>A	TGFBR2 c.382_383delAA
2258	V216I	52.33	male	distal	2	mod	yes	EMAST-MSI+	KRAS c.38G>A	PMS1 c.1015 del A,TGFBR2. c831 del G
2454	A380T	64.46	female	proximal	1	mod	no	EMAST+MSI+		TGFBR2 c.382_383delAA
2842	T340M	67.48	male	rectum	3	mod	no	EMAST-MSI+	APC c.1495C>T	TGFBR2. c831 del G
3796	V216I	54.25	male	proximal	2	mod	no	EMAST+MSI+	KRAS c.35G>A	TGFBR2 c.382_383delAA
4016	T340M	67.71	female	proximal	3	poor	no	EMAST+MSI+	HRASc182AT	TGFBR2 c.831 del G,MSH3 c.1221 del C

Molecular and clinicopathological features of Solitary POLE germline mutation

Sample no.	Germline Variants	Age	Gender	location	TNM	differentiation	LVI	Molecular alterations in tumor		
924	I238F	80.7	Male	Proximal	3	mod	no	EMAST+MSS	POLEc.756 T>A	
1998	K1878C	79.56	Female	Proximal	3	poor	yes	EMAST+MSI+	PIK3CA c.1624G>A， PIK3CA c.1633G>A	POLE c4381del c, c.3591 T>A
2738	Y1351H	70.84	Male	Proximal	2	mod	no	EMAST+MSI+	BRAF c.1799T>A	POLE c3522 G>A, c2135 del A c.4095 C>T
3758	N525D	96.37	Male	Distal	2	mod	no	EMAST-MSI+	KRAS c.35G>A	
3894	A992T	46.18	Female	Rectum	2	mod	no	EMAST-MSI+	KRAS c.436G>A	POLE c.3018 C>T
4104	G433D	36.12	Male	Rectum	3	mod	no	EMAST+MSS	KRAS c.38G>A， NRAS c.181C>A	POLE c.3682 T>C
4266	P2691S	81.42	Male	Rectum	3	mod	yes	EMAST-MSI+	KRAS c .437C>T	POLE c.2894 C>A, c 1275 C>A
4732	N525D	70.46	Male	Rectum	3	mod	yes	EMAST+MSS		
