# Supplementary material for: Patterns of germline and somatic mutations in 16 genes associated with mismatch repair function or containing tandem repeat sequences
Source: Cancer Med. 2019 Nov 25;9(2):476–86. doi: 10.1002/cam4.2702 (PMC6970039; doi:10.1002/cam4.2702)
Supplement: Supplementary file 3 [file CAM4-9-476-s003.pdf]

Table S1: Numbers of all somatic mutation and pathological somatic mutation of these 16 genes panels

	All variants			Pathological variants		
Gene	GM(＋)	GM(－)	*P* value	GM(＋)	GM(－)	*P* value
AXIN1	154.6 ± 0	267.8 ± 121.1	0.188	99.4 ± 15.6	145.6 ± 87.5	0.457
AXIN2	315.5 ± 122.4	258.1 ± 119.0	0.010 [b]	176.7 ± 94.2	139.6 ± 85.0	0.020[b]
BAX	309.0 ± 0	266.6 ± 121.2	0.727	132.5 ± 0	145.3 ± 87.4	0.727
CTNNB1	441.7 ± 0	266.1 ± 120.7	0.148	331.3 ± 0	144.4 ± 86.6	0.032
EPCAM	257.3 ± 158.1	268.8 ± 112.0	0.584	130.8 ± 106.9	148.2 ± 82.6	0.253
EXO1	375.4 ± 180.8	265.4 ± 119.9	0.118	191.4 ± 111.2	144.6 ± 87.0	0.357
MLH1	262.5 ± 89.3	267.6 ± 126.0	0.818	128.1 ± 63.6	148.3 ± 90.6	0.208
MSH2	281.6 ± 111.9	265.1 ± 122.2	0.530	150.9 ± 77.2	144.6 ± 88.5	0.737
MSH3	228.2 ± 54.1	268.4 ± 122.8	0.330	105.5 ± 54.9	146.8 ± 88.0	0.167
MSH6	276.9 ± 165.6	264.8 ± 110.3	0.570	150.1 ± 111.1	144.2 ± 81.9	0.705
PMS1	269.4 ± 91.6	266.7 ± 121.7	0.961	141.3 ± 50.8	145.3 ± 87.9	0.920
PMS2	311.7 ± 155.9	263.0 ± 117.6	0.112	168.8 ± 107.6	143.3 ± 85.4	0.246
POLD1	250.8 ± 97.5	269.0 ± 123.9	0.456	126.9 ± 72.0	147.7 ± 89.0	0.239
POLE	214.9 ± 107.7	270.4 ± 121.3	0.086	120.7 ± 75.9	146.9 ± 87.9	0.073
TGFBR2	263.5 ± 98.2	267.1 ± 122.6	0.914	157.5 ± 83.4	144.3 ± 87.6	0.573

[b]statistically significant difference, $P < 0.05$

[a]GM: germline mutation,
