# Supplementary material for: Patterns of germline and somatic mutations in 16 genes associated with mismatch repair function or containing tandem repeat sequences
Source: Cancer Med. 2019 Nov 25;9(2):476–86. doi: 10.1002/cam4.2702 (PMC6970039; doi:10.1002/cam4.2702)
Supplement: Supplementary file 4 [file CAM4-9-476-s004.pdf]

Table S2: The number of functional solitary gene germline mutation and the number of accompany MMR-related gene germline mutations in patients with either EMAST+ or MSI-H

	No accompany MMR-related gene germline mutation Case No. (%)	Accompany MMR-related gene germline mutation Case No.(%)
AXIN2	11 (31.4)	24 (77.1)
EXO1	0 (0)	3 (100)
MSH3	1 (11.1)	8 (88.9)
PMS1	1 (20)	40 (80)
POLD1	4 (10.5)	26 (86.7)
POLE	8 (53.3)	7 (40.0)
*TGFBR*2	6 (40.0)	9 (60.0)
