# Supplementary material for: Patterns of germline and somatic mutations in 16 genes associated with mismatch repair function or containing tandem repeat sequences
Source: Cancer Med. 2019 Nov 25;9(2):476–86. doi: 10.1002/cam4.2702 (PMC6970039; doi:10.1002/cam4.2702)
Supplement: Supplementary file 5 [file CAM4-9-476-s005.pdf]

Table S3: Clinicopathological features of four cases with rare germline mutation

Mutation Genes	Age	Gender	location	TNM	grade	subtype	molecular alteration in tumors
AXIN1	52	M	proximal	IV	poor	EMAST-MSI+	PIK3CA c.3140A>G,
AXIN1	67	F	proximal	I	well	EMAST-MSI+	KRAS c.35G>A, APC c.4348C>T
BAX	80	F	proximal	I	moderate	EMAST+MSI+	BRAF c.1799T>A, TGFBR2 c.373_374insA
CTNNB1	75	F	proximal	II	poor	EMAST+MSI+	BRAF c.1799T>A, FBXW7 c.1436G>A
